# Surface-Mount Zero-Ohm Jumper Resistor Characterization in High-Speed Controlled Impedance Transmission Lines

**DOI:** 10.3390/s23094472

**Published:** 2023-05-04

**Authors:** Aleksandr Vasjanov, Vaidotas Barzdenas

**Affiliations:** Department of Computer Science and Communications Technologies, Vilnius Gediminas Technical University (VILNIUS TECH), 10223 Vilnius, Lithuania; vaidotas.barzdenas@vilniustech.lt

**Keywords:** 0R resistor, jumper, zero-ohm resistor, zero resistance

## Abstract

Zero-ohm resistors, also known as jumpers, are commonly used in early radio frequency (RF) prototypes as they can help engineers identify the most optimal engineering solution for their system or create application-specific hardware configurations in products. One of the key considerations when using zero-ohm jumpers in RF circuits is the potential for signal loss and interference. Every circuit connection creates a small amount of resistance and impedance, eventually adding up over long distances or in complex circuits. This paper proposes a quantitative characterization summary of standard 0201-, 0402-, 0603-, and 0805-size surface-mount package jumpers, as well as lead-free and lead solder wires, in high-frequency applications by means of time domain reflectometry (TDR) and S-parameter measurements. The typical offset from the target 50 Ω impedance was measured to be around 3 Ω, or 5.8% relative to the measured reference value. According to S-parameter measurement results, no visible impact on attenuation was spotted up to 5 GHz compared to the reference S_21_ curve.

## 1. Introduction

The market for RF components and products, used in a variety of applications including wireless communication, is experiencing rapid growth. The evolution of 5G technology is one of the main drivers of this growth, as it requires more advanced and sophisticated RF components and products to support the higher-frequency bands and bandwidths used by 5G networks. According to a report by the worldwide market research and consulting organization Precedence Research, the market for RF components and products is expected to grow from USD 31.64 billion in 2022 to USD 101.09 billion by 2032, at a compound annual growth rate (CAGR) of 12.32% during the forecast period. This growth is driven by the increasing demand for RF components and products in various end-use industries, such as telecommunications, aerospace and defense, and automotive, among others [1,2].

These advanced technologies often incorporate zero-resistance resistors in prototyping various products, as well as in the final ones. Zero-ohm resistors are very commonly used in early RF prototypes as they can help engineers identify the most optimal engineering solution for their system or reconfigure it, if needed. A jumper is an electrical component used in electronic circuits to connect two points together. In radio frequency (RF) circuits, zero-ohm jumpers are commonly used to connect various components, such as resistors, capacitors, and inductors, between themselves and other integrated circuits. The use of jumpers in RF circuits can have a significant impact on the performance of the circuit, and careful consideration must be given to their incorporation into the design and placement on the printed circuit board (PCB). The placement of these jumpers is critical, as their characteristics, the way they are routed, and proximity to other components can affect the overall performance of the circuit. An important use of zero-ohm jumpers in RF circuits is to create signal paths that bypass certain components in the circuit or make changes to the circuit if an alternative component requires additional external parts (e.g., filters or impedance-matching circuits). Bypassing a component with a jumper is useful when the PCB has more than one assembly configuration for specific tasks. One of the key considerations when using jumpers in RF circuits is the potential for signal loss and interference. Every connection in a circuit creates a small amount of resistance and impedance, which can add up over long distances or in complex circuits. Careful placement and design of jumpers can help minimize these effects, and the use of high-quality components and materials can also help reduce signal loss and interference. When using zero-resistance resistors, it is important to note that they are not completely without resistance. While their impedance is extremely low, it is not zero. As a result, engineers should still take into account any potential impedance when designing their system.

It has also been identified that interest in whether and how zero-ohm jumper resistors impact the performance of RF chains is present in various engineering communities and forums, with most of the queries not providing a definitive and quantitative resolution. Thus, so far, this discussion has been an open topic.

Various research papers and commercial spec-sheets can be found on the topic characterizing passive surface mount devices. The authors of [3] conducted high-frequency measurements on thin-film resistors, analyzing their impedances up to 40 GHz, although the smallest resistance value was 50 Ω. The scope of the latter study included 50 Ω, 100 Ω, 200 Ω, 500 Ω, and 1 kΩ values. Solder joint research under direct current (DC) loads with 0402 and 0603 surface mount resistors was conducted in [4]. The authors of [5] proposed an inexpensive method for characterizing passive components in high-frequency applications, while the authors of [6] presented equivalent circuits of small-size chip resistors up to 50 GHz, although the latter included quartz 0102 resistors with a lowest value of 10 Ω. Zero-ohm resistors are mentioned as a possible alternative to a ferrite bead because they exhibit some impedance due to component package parasitics [7]. Resistor package parasitics and the use of surface-mount devices in RF circuits is mentioned in [8]. Commercial application notes [9,10] provide frequency responses for various value resistors and describe their use in RF devices, but the smallest resistance value goes down only to 5 Ω.

To the best of the authors’ knowledge, surface mount zero-ohm jumper resistor impact on controlled impedance microstrips has not been characterized. Thus, this paper proposes a quantitative characterization of surface-mount zero-ohm resistors, encapsulated in various packages and manufactured using different processes, in a high-frequency 50 Ω impedance transmission line.

The paper is organized as follows: the introduction is followed by Section 2, which provides the motivation for this research. Section 3 discusses the differences in zero-ohm resistor technologies and packages. Section 4 presents the device under test (DUT) setup and measurement results. The results presented in this paper are then summarized in Section 5, with references provided afterwards. The Appendix A include *S*-parameter and time domain reflectometry (TDR) measurement result extended plots, as well as a table with DUT part descriptions.

## 2. Motivation

Zero-ohm resistors, also known as jumpers, have become increasingly popular in recent years for use in the configuration of antennas and various radio frequency (RF) circuits, as reported in several scientific studies [11,12,13]. These components, functioning as short circuits, offer a flexible means to interconnect various components and bypass elements within a circuit, thereby enabling greater design and testing versatility. Despite the fact that zero-ohm resistors possess extremely low resistance values, their impedance is not negligible and thus can affect the electrical characteristics of the circuit. Specifically, the impedance of a zero-ohm resistor contains capacitive or inductive components that require careful consideration when configuring circuits that employ these resistors.

**Figure 1:**
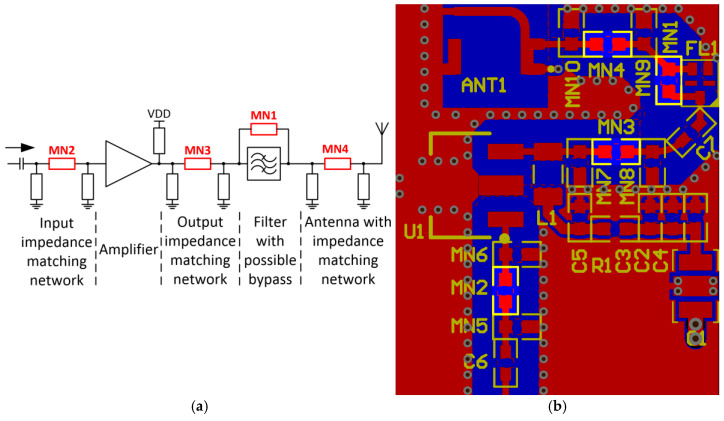
Zero-ohm jumper resistors in high-speed analog transmission lines: simplified RF front-end block diagram (**a**), RF front-end PCB layout (**b**).

A practical example of a RF front-end chain with possible reconfiguration is presented in Figure 1. Figure 1a shows an amplifier with external input and output impedance matching networks, followed by a filter, which is connected to an antenna. The possible positions of jumper resistors are components with designators MN1-MN4. Depending on the type, a broadband gain stage or a narrow-band amplifier can be implemented to meet the target goal. In the case of a broadband gain stage, external impedance-matching networks are usually not required. If the selected initial amplifier contains an alternative part with the same package and pin-out, parts can be swapped during the lifecycle of the device (e.g., part shortages, end-of-life or specific requirements). Even if the alternative part is compatible with the current amplifier land pad configuration, external impedance matching networks are sometimes required; thus, they have to be included in the initial design.

Moving on through the presented chain in Figure 1a, filters are tied to a specific frequency band (e.g., LTE, GSM, etc.), and if the amplifier configuration prior to that changes to another specific frequency, the filter might be changed to another one or simply bypassed using an MN1 jumper. Different frequency range filters often have different packages or pinouts, and if a filter selection by means of changing a jumper resistor is included, there will be two zero-ohm jumpers instead of a single MN1. Solid-state RF switches are always an option, but if the amplifier is initially configured to a specific band, there is no need for this pricy solution.

The antenna, which is also frequency-specific, is the last block in Figure 1a. Depending on the type (on-board or external), it can require an impedance-matching network. If it does not, the series component MN4 becomes a jumper. A possible layout of the block Figure 1a diagram with highlighted jumper resistors is provided in Figure 1b. All in all, modern devices can have hardware configurations dictated by specific requirements and multiple jumpers can be included in high-frequency chains, which require maintaining a specific impedance. The latter front-end is just an example which can be extrapolated to other similar high-frequency chains. Because zero-ohm jumpers are enclosed in different surface-mount packages and fabricated differently, resulting in various types, it is not intuitive which one introduces a smaller impact (losses and impedance discontinuity) in the chain, mitigating the overall performance.

## 3. Differences in Zero-Ohm Resistor Technologies and Packaging

Although different types of zero-ohm resistors enclosed in the same package should exhibit almost identical characteristics, the nuances in their fabrication ensure their own advantages and disadvantages.

Thin-film resistor technology depends on the deposition of a thin metallic layer on a ceramic substrate. Resistors built using this technology possess enhanced resistance for a given area, and thus this type of resistors are reasonable and save a lot of space. However, the film is susceptible to failure owing to high temperatures, chemical contamination, and water vapor.

Resistors fabricated using thick-film technology are formed by applying a resistive metallic paste on the base. They offer improved resistance per given area and are cost-efficient as compared to other types of resistors, such as wire-wound resistors. Their frequency is similar to foil and thin-film resistors. However, they are comparatively noisier than other types. Notwithstanding their disadvantages, they are extensively used in circuit units that need less accuracy and permanency.

Foil resistors are formed by applying a metal foil to a ceramic substrate which is photo-etched with a resistive design. This procedure develops a resistor with the advantageous features of improved stability, reduced capacitance, non-inductance, and reduced noise, without forgoing speed and precision. Hence, they are widely in demand from various electronics industries [14].

Metal element resistors are usually used as current-sense elements in high-power applications.

**Figure 2:**
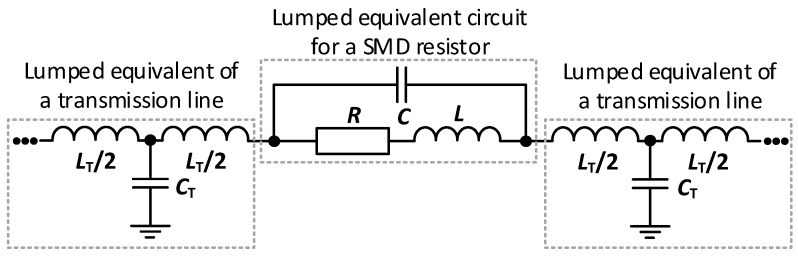
Equivalent lumped circuit model for a resistor connected to a transmission line.

Figure 2 presents the simplest, but the most common, lumped circuit model for a passive component with parasitic parameters. Apart from the main parameter for the SMD resistor, its resistance *R*, parasitic inductance *L* and capacitance *C* are also included due to the package. *C*_T_ and *L*_T_ describe the lumped equivalent of a transmission line. According to [15], the lumped model *L* and *C* components presented in Figure 2 are in the range of pH and pF accordingly, but they still affect the overall impedance.

## 4. DUT Setup and Measurement Results

**Figure 3:**
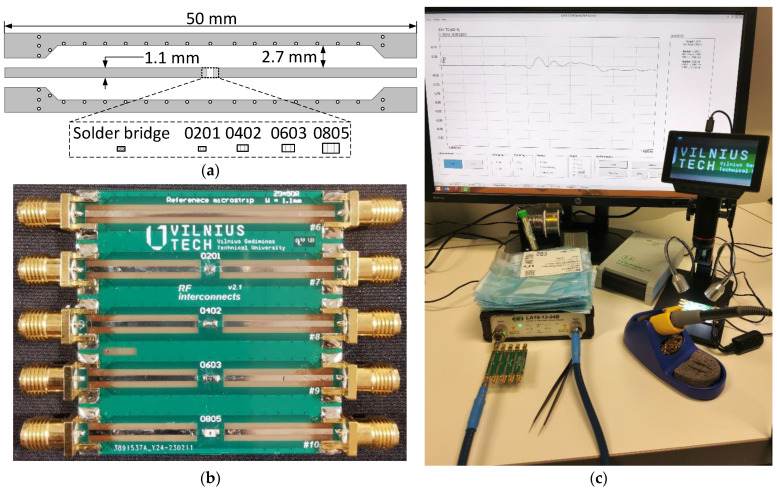
Zero-ohm jumper resistor measurement setting: single RF trace parameters (**a**), fabricated PCB (**b**), DUT test-bench (**c**).

The device under test (DUT) contains separate 50 mm long microstrip lines with a target impedance of *Z*_0_ = 50 Ω, as shown in Figure 3a. The manufactured PCB is presented in Figure 3b and contains a reference microstrip and four microstrips with a place to mount a jumper in the center. The trace width was selected to be equal to the width of the 0805-size SMD jumper, which is the largest component presented in this paper. This is used to remove the effect of the footprint pads, which would be wider than a typical *Z*_0_ = 50 Ω microstrip in a multilayer PCB, on the overall impedance. As a result, all observed changes in *S*-parameter and TDR responses compared to the reference microstrip measurement data are due to the introduced zero-ohm jumper. Resistors were soldered using S-Sn63Pb37E solder paste and the soldering area was cleaned using isopropyl alcohol before each measurement for the purity of the experiment.

Measurements and experimental analysis were performed using a calibrated 8.5 GHz bandwidth LA19-1304B VNA. The fabricated DUT PCB was connected directly to the VNA, avoiding the use of additional cables that introduce additional insertion losses and possible reflections for *S*_11_ and TDR measurements. A 50 Ω calibration load was connected to the other end of the DUT during *S*_11_ and TDR measurements. During *S*_21_ response measurement, the DUT input was directly connected to the VNA, whereas the second end was connected to the second port of the VNA using a low-loss Sucoflex 126EA test lead/cable, as shown in Figure 2c. The taken measurement steps for a single cycle are as follows:

(1) Solder the selected size and part number zero-ohm resistor on the appropriate transmission line and clean the flux residue with isopropyl alcohol;
(2) OSLT-calibrate the VNA for *S*-parameter measurements;
(3) Connect the DUT PCB input directly to the VNA and load the second end of the transmission line with a high-frequency 50 Ω calibration load;
(4) Measure *S*_11_ parameter;
(5) Remove the high-frequency 50 Ω calibration load from the second end of the transmission line and connect it to the second port of the VNA using low-loss Sucoflex 126EA cable;
(6) Measure *S*_21_ parameter;
(7) OSL-calibrate the VNA for TDR measurements;
(8) Connect the DUT PCB input directly to the VNA and load the second end of the transmission line with a high-frequency 50 Ω calibration load;
(9) Measure TDR response.

**Figure A1:**
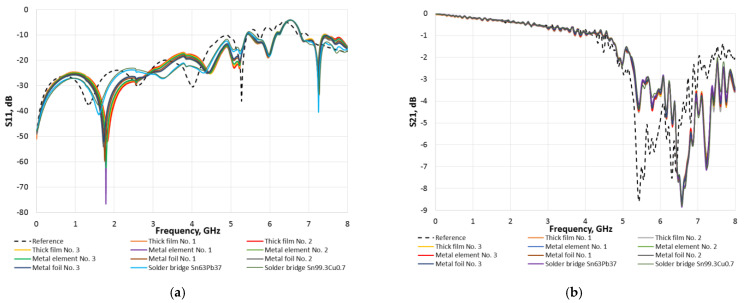
0201 surface-mount package zero-ohm jumper scattering parameter measurement results: *S*_11_ (**a**), *S*_21_ (**b**).

**Figure A2:**
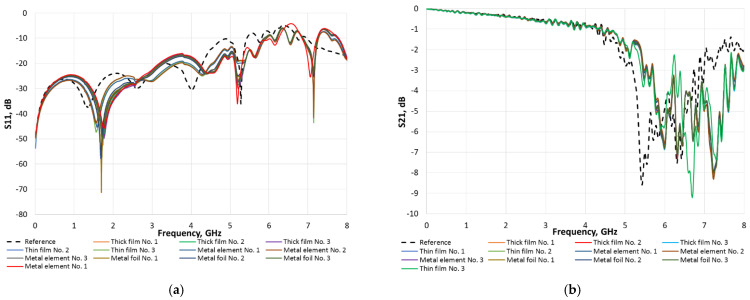
0402 surface-mount package zero-ohm jumper scattering parameter measurement results: *S*_11_ (**a**), *S*_21_ (**b**).

**Figure A3:**
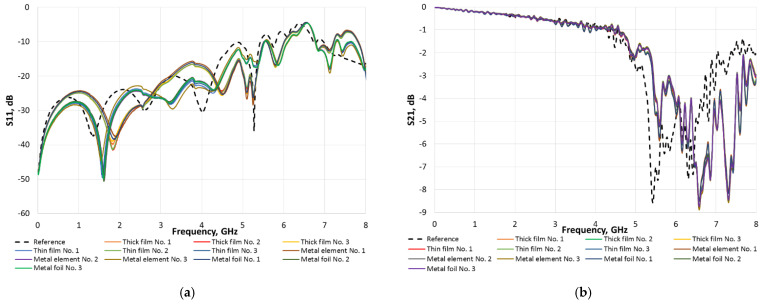
0603 surface-mount package zero-ohm jumper scattering parameter measurement results: *S*_11_ (**a**), *S*_21_ (**b**).

**Figure A4:**
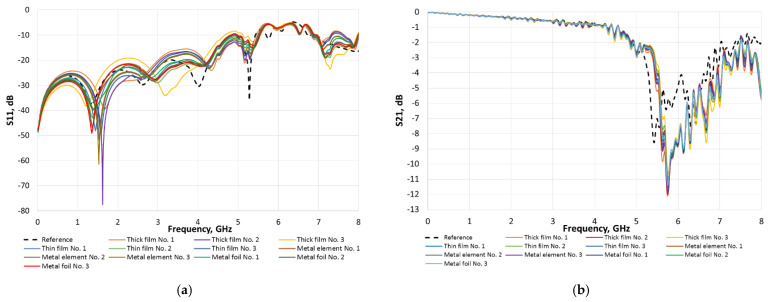
0805 surface-mount package zero-ohm jumper scattering parameter measurement results: *S*_11_ (**a**), *S*_21_ (**b**).

**Figure A5:**
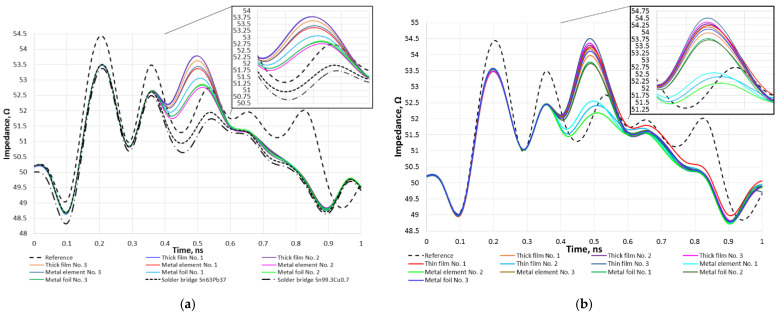
Full TDR responses for 0201 (**a**) and 0402 (**b**) surface-mount package zero-ohm jumpers.

**Figure A6:**
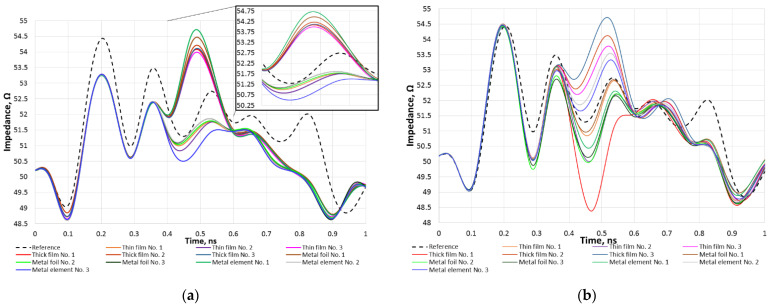
Full TDR responses for 0603 (**a**) and 0805 (**b**) surface-mount package zero-ohm jumpers.

Zero-ohm jumpers were selected based on two major parameters—package size and jumper type. In the scope of this paper, only standard 0201-, 0402-, 0603-, and 0805-size packages were selected, as they are the most common in modern high-speed analog and digital devices. The 01005-size package was not included as these components are problematic to solder by hand and only have a handful zero-ohm part of alternatives, whereas 1206 and larger jumpers are not likely to be used in modern high-speed communication interconnects. The jumper type defines the technology and materials for a specific part, so the most common types for all packages were included, such as thick film, thin film, metal element, and metal foil. The part types and their descriptions are listed in Appendix A Table A1. Manufacturer part numbers are not listed in order not to highlight any specific manufacturer or part. Each curve presented in Figure 3, as well as in Appendix A Figure A1, Figure A2, Figure A3, Figure A4, Figure A5 and Figure A6, is an average of 5 samples of the same part. Moreover, measurements of basic solder bridges using lead-free Sn99.7Cu0.7 SW26/2.5% and lead S-Sn63Pb37E solder wires were also included as a possible jumper solution. The latter solder bridges were soldered over 0201 component solder pads.

**Figure 4:**
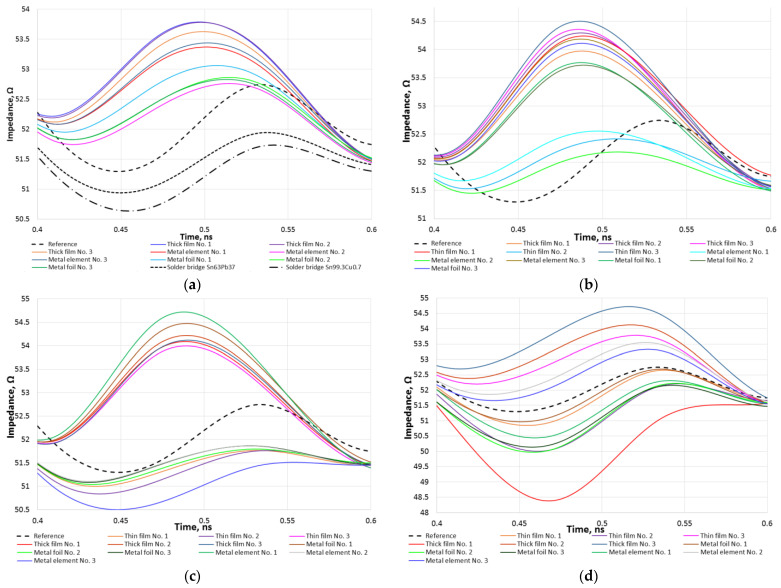
Zero-ohm jumper resistor TDR measurement results: 0201 package and solder bridge (**a**), 0402 package (**b**), 0603 package (**c**), 0805 package (**d**).

Figure 4 presents the TDR measurement results for each package type at the center of the microstrip where the zero-ohm jumpers are assembled. Full TDR and *S*-parameter measurement plots are presented in the Appendix A in Figure A1, Figure A2, Figure A3, Figure A4, Figure A5 and Figure A6. 

In all Figure 4 plots, the long-dashed black curve corresponds to the reference microstrip with no jumper TDR measurements, whereas other curves represent various types of jumper resistor measurement results. The 0201 package size jumper measurement results in Figure 4a additionally contain short-dashed and dash–dot black curves, which represent solder bridge with and without lead responses accordingly. All aforementioned plots contain multiple curves, which might be hard to distinguish, but the main goal in providing responses in this format is to visually show how small the differences between various jumper resistor type impedance shifts are.

After analyzing the measurement results for the 0201 package, presented in Figure 4a, it can be said that both solder bridges are up to 1 Ω below the reference impedance curve. However, all types of zero-ohm jumper resistors show an increase in impedance ranging from 0.5–2.3 Ω. The pink curve corresponds to a metal element part and is closest to the reference, whereas the blue curve corresponds to a thick-film part and has the farthest value.

Figure 4b presents the measurement results for the 0402 surface-mount jumpers, divided into two groups of curves: those closest to the reference curve, and all others. The first group consists of two metal element parts (aqua blue and light green curves) from different manufacturers, and a thin-film part (light blue). The second group is comprised of all other types of jumper measurements, which exhibit a 1.5–2.2 Ω increase in impedance compared to the reference results. The dark green curve represents a metal foil part with the lowest increase in impedance, while the dark blue curve represents a thin-film part with the highest increase.

Figure 4c shows the measurement results for the 0603 package size jumpers, which can be divided into two groups, based on undershoot and overshoot impedance values compared to the reference measurements. In the group of curves with an undershoot impedance value, a metal element part (dark blue curve) refers to a 1 Ω lower impedance than the reference measurement, whereas a metal element part from a different manufacturer (dark green curve) is the closest to the reference curve and lower by around 0.5 Ω. Other parts in this group include two metal foil parts from different manufacturers (green and red curves), as well as a thin-film (purple curve) one. The thin-film part on the lower side (pink curve) and a metal element (green curve) part are on different ends of the group of curves which overshoot the reference measurement curve. The offset is around 1.7 Ω and 2.5 Ω accordingly.

Figure 4d contains the 0805 package size surface-mount jumper measurement results. The scatter in the measurements for all parts spread in the range of a 4 Ω overshoot, corresponding to a thick-film jumper (dark blue curve), and a 3.5 Ω undershoot, corresponding to different a thick-film jumper (red curve).

Summarizing the curves presented in Figure 4, the 0201 size surface mount zero-ohm jumpers mostly overshoot the reference measurements regardless of the type. Solder bridges over 0201 solder pads provide an impedance which is slightly below the reference. The 0402- and 0603-size surface mount jumpers are different from 0201 jumpers, as in both cases some parts show a value smaller than the reference impedance value, while others show a larger one. The 0805-size surface mount jumper impedance measurements form a more or less even spread of possible impedance values around the reference. In all cases, no tendency of one type of jumper (e.g., metal foil) from different manufacturers showing near identical results was identified. The same part type from different manufacturers can shift the impedance both ways, either to the overshoot (with a dominant inductance in the impedance) or the undershoot (with a dominant capacitance in the impedance) regions.

**Figure 5:**
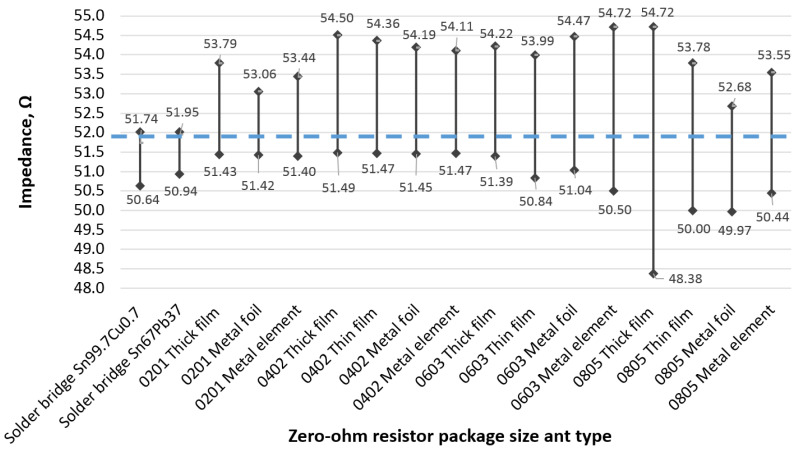
Zero-ohm jumper resistor TDR measurement summary.

Figure 4 presents TDR measurement curves, each of which is a mean of 5 experiments, and non-intuitive and raw data. All measurement results have been processed and presented in Figure 5, which shows the possible jumper resistor impedance deviations from the reference value. It can be seen that only the 0805 thick-film resistors showed the largest possible window of impedance values, which is equal to 6.34 Ω, or 11.5% relative to the measured target reference. The 0402 and 0603 surface-mount package size jumpers provide a similar scatter of possible impedances equal to around 3 Ω, or 5.8% relative, and are most likely to overshoot the reference value. The 0201-size jumpers show the smallest impedance scatter with metal foil type having only a 1.5 Ω, or 2.9% relative, offset and more likely to the larger side. From the results in Figure 5, it can be concluded that zero-ohm jumpers exhibit dominant inductive parasitic parameters, which are included in Figure 4, in high-frequency transmission lines [16]. As for the solder bridges, both lead-free and lead solder connections show the smallest offset of just 1 Ω, or 1.9% relative, with a tendency of undershooting the reference.

According to *S*-parameter measurements, presented in Appendix A Figure A1, Figure A2, Figure A3 and Figure A4, the microstrip with jumper resistors, regardless of the package size and type, do not introduce any visible attenuation up to 5 GHz compared to the reference *S*_21_ curve. The *S*_21_ curve with jumper resistors above 5 GHz is similar in shape, but is shifted by around 600 MHz to the upper range. *S*_11_ measurement results are also similar to the reference measurement results in amplitude with minor shifts in frequency.

## 5. Conclusions

Zero-ohm jumper resistors are commonly used in early RF device prototypes, as well as when creating possible hardware reconfiguration capabilities during the assembly stage of final products. This paper proposes a quantitative characterization summary of jumpers in standard 0201-, 0402-, 0603-, and 0805-size packages, as well as lead-free and lead solder wires, in order to determine their effect on the impedance of a high-frequency digital or analog chain. The results of 5 experiments with each of the 45 different parts from various manufacturers and 2 types of solder showed that, regardless of the jumper types and package sizes, the typical offset from the target 50 Ω impedance was around 3 Ω, or 5.8% relative. Both types of solder bridges performed similarly, with an offset of just 1 Ω, or 1.9% relative, with a tendency of undershooting the reference. According to the *S*-parameter measurement results, no visible impact on attenuation was spotted up to 5 GHz compared to the reference *S*_21_ curve. Zero-ohm jumper resistors above 5 GHz had a similar shape response to that of the reference microstrip, but were shifted by around 600 MHz to the upper range. The *S*_11_ measurement results were also similar in amplitude to the reference measurement results with minor shifts in frequency.

## Data Availability

Not applicable.

## Appendix A

Appendix A provides extended TDR and S-parameter responses for the DUT. They include measurements for the 0201-, 0402-, 0603-, and 0805-size surface-mount zero ohm resistor packages assembled on the carrier PCB with a target impedance of 50 Ω. A table describing zero-ohm resistors in the scope of the research is also given without highlighting specific part numbers or manufacturers.

**Table A1:** Zero-ohm jumper types used and their description.

No.	Package	Quantity	Type	Description
1	0201	5	Thick Film	RES SMD 0 OHM JUMPER 1/20W 0201
2				RES 0 OHM JUMPER 1/20W 0201
3				RES SMD 0 OHM JUMPER 1/20W 0201
4			Metal Element	RES 0 OHM JUMPER 1/10W 0201
5				RES 0.01 OHM 1% 1/5W 0201
6				RES 0 OHM JUMPER 0201
7			Metal Foil	RES 0.02 OHM 1% 1/4W 0201
8				RES 0.05 OHM 1% 1/10W 0201
9				RES 0.01 OHM 1% 1/4W 0201
10	0402		Thick Film	RES SMD 0 OHM JUMP 1/16W 0402
11				RES 0 OHM JUMPER 1/16W 0402
12				RES SMD 0 OHM JUMPER 1/10W 0402
13			Thin Film	RES 0.33 OHM 1% 1/6W 0402
14				RES 0.22 OHM 1% 1/6W 0402
15				RES 0.1 OHM 1% 1/6W 0402
16			Metal Element	METAL CURRENT SENSOR-LOW T.C.R C
17				RES SMD 0 OHM JUMPER 1/5W 0402
18				RES 0 OHM JUMPER 0402
19			Metal Foil	RES 0 OHM JUMPER 1/8W 0402
20				RES 0.05 OHM 1% 1/16W 0402
21				RES 0.024 OHM 0.25 W 1% 0402 SMD
22	0603		Thick Film	RES 0 OHM JUMPER 1/10W 0603
23				RES 0 OHM JUMPER 1/10W 0603
24				RES 0 OHM JUMPER 1/10W 0603
25			Thin Film	RES 0.01 OHM 1% 1/4W 0603
26				RES 0 OHM JUMPER 1/10W 0603
27				RES SMD 0 OHM JUMPER 0603
28			Metal Element	RES 0 OHM JUMPER 0603
29				RES 0 OHM JUMPER 1/2W 0603
30				RES 0 OHM JUMPER 0603
31			Metal Foil	RES 0.03 OHM 0.5 W 1% 0603 SMD
32				RES 0 OHM JUMPER 1/4W 0603
33				RES 0.04 OHM 1% 1/10W 0603
34	0805		Thick Film	RES 0 OHM JUMPER 1/8W 0805
35				RES SMD 0 OHM JUMPER 1/8W 0805
36				ULTRA-LOW OHMIC CHIP RESISTORS F
37			Thin Film	RES 0 OHM JUMPER 1/8W 0805
38				RES 0.05 OHM 5% 1/4W 0805
39				RES 0.01 OHM 5% 1/4W 0805
40			Metal Element	RES 0 OHM JUMPER 1/2W 0805
41				RES 0.015 OHM 1% 1/2W 0805
42				RES 0.01 OHM 1% 1/2W 0805
43			Metal Foil	RES 0.012 OHM 0.5 W 1% 0805 SMD
44				RES 0.01 OHM 1% 3/4W 0805
45				RES 0 OHM JUMPER 1/2W 0805
46	Solder bridge	-	Lead-free	Sn99.7Cu0.7 SW26/2.5%
47			With lead	S-Sn63Pb37E
